# Deletion of GIT1 Impacts eNOS Activity To Aggravate sFlt-1–Induced Preeclampsia Phenotype in Mice

**DOI:** 10.1534/g3.118.200509

**Published:** 2018-08-22

**Authors:** Shenghong Zhang, Cuili Zou, Qiaoqin Zhang

**Affiliations:** Heze Municipal Hospital, Heze City 274000, Shandong Province, China

**Keywords:** preeclampsia, endothelial nitric oxide synthases (eNOS), G-protein-coupled Receptor Kinase Interactor-1 (GIT1), NO production, urinary albumin excretion (UAE), blood pressure (BP)

## Abstract

Preeclampsia, a serious multisystem disorder specific to human pregnancy, remains a considerable burden of disease worldwide. Reduced nitric oxide bioavailability is proved to be crucial in the maternal and fetal pathophysiology of preeclampsia. G-protein-coupled Receptor Kinase Interactor-1 (GIT1) is a novel endothelial nitric oxide synthases (eNOS) interactor mediator. The aim of this paper is to investigate the effect of GIT1 on preeclampsia. Blood pressure (BP) was measured using a carotid catheter-calibrated eight-chamber tail-cuff system (CODA) at the same time daily. Urinary albumin excretion (UAE) was determined using Albuwell-M kits (Exocell Inc) and creatinine clearance (CCr) was determined by measuring urinary creatinine concentration with tandem liquid chromatography–mass spectrometry. The release of nitrite was analyzed to detect nitric oxide (NO) production using a Sievers Chemiluminescence NO Analyzer. NOS activity was examined by measuring the conversion of ^3^H-labeled l-arginine to ^3^H-labeled l-citrulline. BP was significantly increased in GIT1^−/−^ mice with or without sFIT-1 treatment. In addition, GIT1−/− mice possessed higher UAE and lower CCr. Depletion of GIT1 impedes the NO production and placenta eNOS activity. Additional GIT1 attenuates sFlt-1-induced preeclampsia phenotypes. Our findings suggest that GIT1 significantly extenuates the sFlt-1-induced preeclampsia phenotypes by inhibiting eNOS activity, indicating a crucial role of GIT1 in the progression of preeclampsia.

Preeclampsia is a pregnancy-specific disorder traditionally diagnosed by increased blood pressure (greater than 90 mmHg diastolic or 140 mmHg systolic) and proteinuria, commonly affects approximately 3–5% of pregnancies (Mol *et al.* 2016). Especially in less developed countries, preeclampsia accounts for the second leading direct cause of maternal death, and remains one of the main causes of fetal and neonatal mortality (Gidlof and Nisell 2010). In severe diseases, kidney dysfunction, red blood cell breakdown, impaired liver function, visual disturbances, swelling, shortness of breath due to fluid in the lungs, or a low blood platelet count may occur (American College of Obstetricians and Gynecologists and Task Force on Hypertension in Pregnancy 2013). However, the pathogenetic mechanisms of preeclampsia are still not yet fully elucidated, putting limits to efficacious treatments.

Nitric oxide (NO), initially recognized as the endothelium-derived relaxing factor, is the chief vasodilator substance generated by the endothelium in response to various chemical and mechanical stimuli (Johal *et al.* 2014). By activating soluble guanylate cyclase (sGC), NO causes the relaxation of vascular smooth muscle cells, which in turn results in the activation of cGMP-dependent protein kinases and an increase in intracellular cyclic guanosine 3′,5′-monophosphate (cGMP) (Buhimschi *et al.* 1998). As a paracrine and autocrine signaling molecule, NO is synthesized from l-arginine by nitric oxide synthases (NOS), which is a family of calcium–calmodulin-dependent enzymes (Conrad and Davis 1995). In mammals, NO is mediated by endothelial NOS (eNOS) and neuronal NOS (nNOS) (Shaamash *et al.* 2001). It has been found that reduction in the bioavailability of NO is a key feature of endothelial dysfunction in preeclampsia (Brennecke *et al.* 1997). Moreover, the depletion of eNOS in mice result in high BP, decreased production of NO, hyperlipidemia, and insulin resistance (Orange *et al.* 2003). Previous studies also show that lack of eNOS exacerbates the preeclampsia–like phenotype induced by overexpression of sFlt-1 in nonpregnant female mice (Li *et al.* 2012).

G-protein-coupled Receptor Kinase Interactor-1 (GIT1) is a GTPase-activating protein for the ADP-ribosylation factor family of small GTP-binding proteins, which connects the signaling proteins to distinct cellular locations (Premont *et al.* 2004). Recent studies have shown that GIT1 not only functions as a scaffolding protein, but also possess intrinsic signaling abilities (Schmalzigaug *et al.* 2007). In addition, it has been demonstrated that GIT1 serves as a novel eNOS interactor modulating after liver injury, suggesting that it plays an important role in regulating the biological function of eNOS (Liu *et al.* 2012). These findings indicate that GIT1 might be a crucial mediator in preeclampsia progression.

In this study, we aimed to understand the role of GIT1 in sFlt-1–induced preeclampsia phenotype in pregnant mice and to elucidate the underlying mechanisms.

## Materials and Methods

### Animals

Animals were purchased from Nanjing Animal Model Institute (Nanjing, China). All animal experiments in this study were conducted in accordance with the International Animal Care and Use Committee guidelines of Heze Municipal Hospital. Pregnant C57BL/6 mice (WT and GIT1^−/−^, embryonic day 13) were injected into the tail veins with 3×10^9^ PFU of adenovirus to overexpress sFlt-1 (sFlt-1) or adenovirus encoding murine Fc protein (control) at equivalent doses. To rule out nonspecific effects of adenovirus, Ad Fc was used as a control.

### Measurement of urinary albumin excretion (UAE)

Urinary albumin was determined using Albuwell-M kits (Exocell Inc, Philadelphia, PA) according to the manufacturer’s instructions.

### Measurement of creatinine clearance (CCr)

CCr was determined by measuring plasma and urinary creatinine concentration with the method developed before using tandem liquid chromatography–mass spectrometry (Takahashi *et al.* 2007).

### Measurement of BP

A computerized tail-cuff system was used for measuring BPs on unanesthetized, restrained mice (Krege *et al.* 1995). Continuous recording of the BPs of was performed by radio telemetry.

### Nitric Oxide Measurement

The release of nitrite (the stable breakdown product of NO) was detected to assess NO production using a Sievers Chemiluminescence NO Analyzer (Sievers Instruments, Inc., Boulder, CO) according to the manufacturer’s instructions.

### NOS Activity Assays

NOS activity was assessed by measuring the conversion of ^3^H-labeled l-arginine to ^3^H-labeled l-citrulline as previously described (García-Cardeña *et al.* 1998) according to the manufacturer’s instructions (Cayman Chemical Co., Ann Arbor, MI).

### Statistical analysis

All data were shown as the mean ± SD. Differences between samples were analyzed using the one or two-way ANOVA analysis followed by a *post hoc* test. Statistical significance was accepted at *P* < 0.05.

### Data availability

The authors state that all data necessary for confirming the conclusions presented in the article are represented fully within the article.

## Results

### BP was significantly increased in GIT1−/− mice with or without sFIT-1 treatment

**Figure 1:**
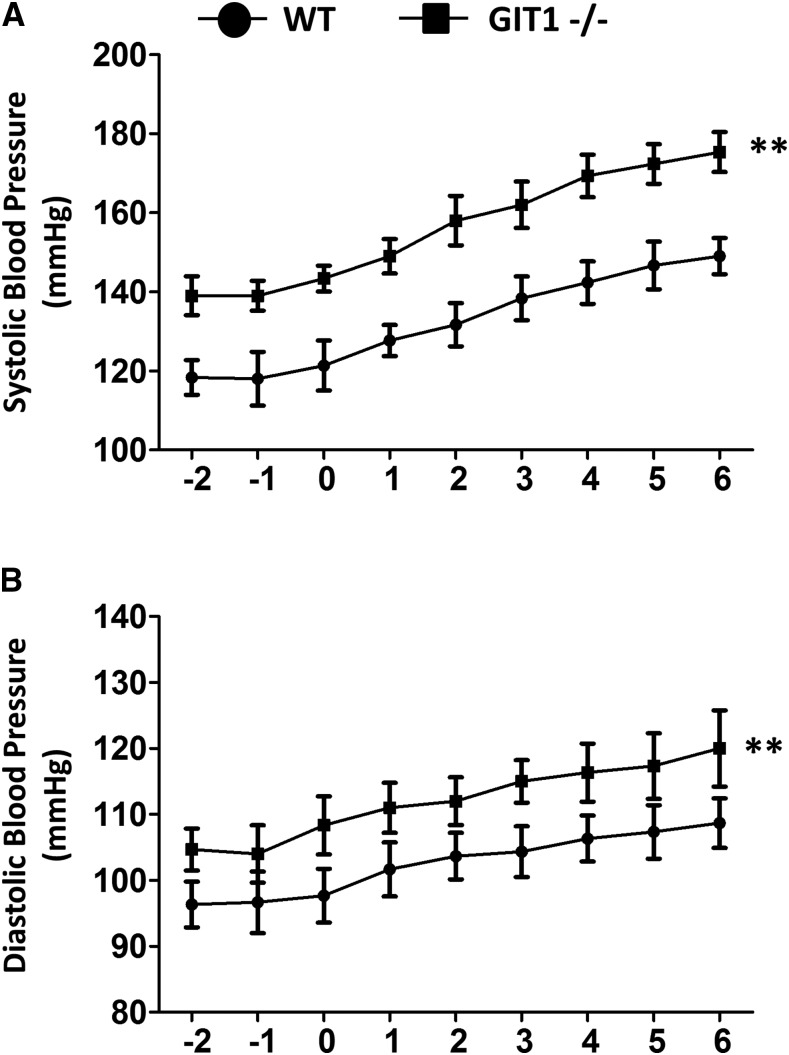
Blood pressures were increased in GIT1^−/−^ mice with or without treated with sFIT-1. (A) Systolic blood pressure (B) Diastolic blood pressure. The tests were started at day 2 (-2) before administration of sFlt-1 and finished at day 6 (6) after administration of sFlt-1, day 0(0) was the day administrated with sFlt-1. N = 6 mice in each experimental group. **P < 0.01 *vs.* WT mice.

Adenovirus (Ad sFlt-1, 3×10^9^ PFU) was injected into pregnant C57BL/6 mice (GIT1−/− and WT, embryonic day 13) to induce preeclampsia. Since hypertension is a major criterion for diagnose of preeclampsia, we applied telemetry to measure BP at the aortic arch. BP were monitored at the same time daily started at day 2 (-2) before administration of sFlt-1 and finished at day 6 (6) after administration of sFlt-1. As shown in Figure 1A and B, sFlt-1 virus increased SBP and DBP of both GIT1−/− and WT mice. Notably, both SBP and DBP were significantly increased in GIT1−/− mice with or without treated with sFIT-1, indicating that the influence of GIT1 on BP was independent of sFlt-1 treatment.

### GIT1^−/−^ mice possess higher urinary albumin excretion and lower creatinine clearance

**Figure 2:**
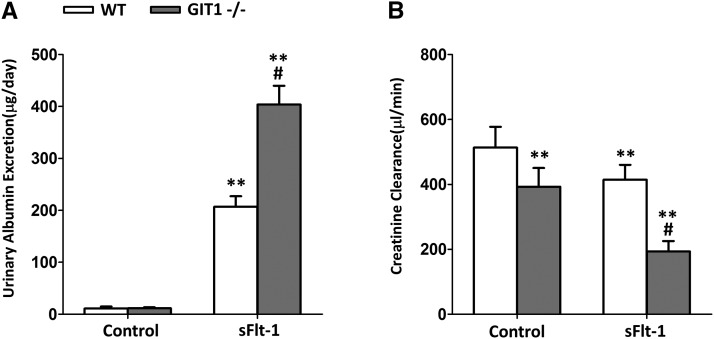
Renal function was impacted in GIT1^−/−^ mice. (A) Urinary Albumin Excretion (B) Creatinine Clearance. The tests were done at day 6 after administration of sFlt-1/Fc(control). N = 6 mice in each experimental group. **P < 0.01 *vs.* WT/control mice, #*P* < 0.01 *vs.* WT/ sFlt-1 mice.

We next examined the UAE and CCr in GIT1^−/−^ and WT mice. As indicated in Figure 2A, daily UAE was not changed in mice of each genotype when treated with control virus (Ad Fc). SFlt-1 virus significantly increased the UAE in both WT and GIT1^−/−^ mice. In addition, the increased sFlt-1 and lack of GIT1 synergistically enhanced the level of UAE (*P* < 0.01). Besides, GIT1 depletion remarkably decreased CCr in WT and GIT1^−/−^ mice (Figure 2B). Again, there was synergistic interaction between sFlt-1 and lack of GIT1, since a greater extent of CCr reduction was observed in GIT1^−/−^ mice when treated with sFlt-1. Thus, the lack of GIT1 and increased sFlt-1 synergistically exacerbates the increase in UAE and the decrease in CCr.

### Depletion of GIT1 impedes the NO production and placenta eNOS activity

**Figure 3:**
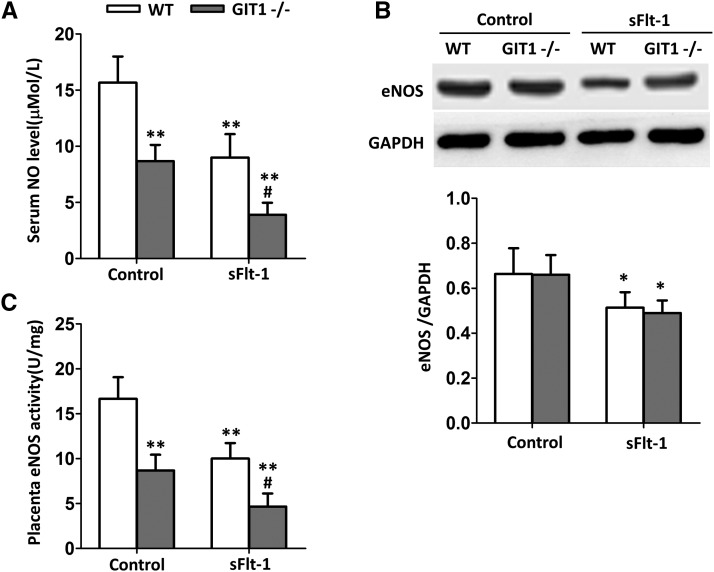
Serum NO level and placenta eNOS activity were impacted in GIT1^−/−^ mice. (A) Serum NO level (B) Placenta eNOS expression (C) Placenta eNOS activity. The tests were done at day 6 after administration of sFlt-1/Fc (control). N = 6 mice in each experimental group. *P < 0.05, **P < 0.01 *vs.* WT/control mice, #*P* < 0.01 *vs.* WT/ sFlt-1 mice.

Reduction in the bioavailability of NO has proved to be a critical feature of endothelial dysfunction in preeclampsia. As shown in Figure 3A, compared with the WT mice, serum NO level was decreased in GIT1^−/−^ mice when treated with Fc, and a more significant decline was detected in GIT1^−/−^ mice when treated with sFlt-1 (*P* < 0.01). Since eNOS is a key factor monitoring the NO synthesis, we next examined the expression level of eNOS in WT and GIT1^−/−^ mice with or without sFlt-1 treatment by western blot. As shown in Figure 3B, sFlt-1 significantly decreased the eNOS level both in the WT and GIT1^−/−^ mice. However, depletion of GIT1 did not impact the expression level of eNOS (Figure 3B). To further clarify the roles of GIT1 and sFlt-1 in regulating the NO production, placenta eNOS activities were evaluated. Consistent with the results shown in Figure 3A, depletion of GIT1 significantly decreased the eNOS activities with or without sFlt-1 treatment (Figure 3C). Moreover, there was a synergistic effect between sFlt-1 and lack of GIT1 on suppressing the placenta eNOS activities (Figure 3C). Thus, we concluded that GIT1 depletion inhibits the NO synthesis by suppressing the activity, instead of suppressing the protein level of eNOS.

### Additional GIT1 attenuates sFlt-1-induced preeclampsia phenotypes

**Figure 4:**
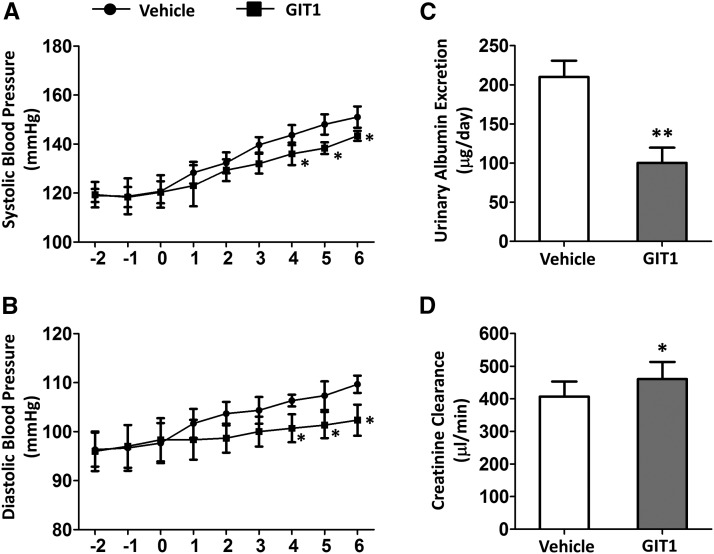
Additional GIT1 attenuated sFlt-1-induced preeclampsia phenotype in WT mice. (A) Systolic blood pressure (B) Diastolic blood pressure. The tests were started at day 2(-2) before administration of sFlt-1 and finished at day 6(6) after administration of sFlt-1, day 0(0) was the day administrated with sFlt-1, day 1 was the day administrated with GIT1. (C) Urinary Albumin Excretion (D) Creatinine Clearance. The tests were done at day 6 after administration of sFlt-1. Vehicle group was WT mice treated with sFlt-1, GIT1 group was WT mice treated with sFlt-1 and GIT1. N = 6 mice in each experimental group. *P* < 0.05,**P < 0.01 *vs.* Vehicle mice.

We overexpressed GIT1 in pregnant C57BL/6 mice by injection of adenovirus to further identify the effects of GIT1 on preeclampsia. As shown in Figure 4A and B, the SBP and DBP were decreased in a time-dependent manner when treatment with additional GIT1, reversing the enhancement in BP induce by sFlt-1. Significant differences were observed on the fourth day after the stimulation of sFlt-1 (*P* < 0.05). In addition, UAE was significantly decreased with additional expression of GIT1 (Figure 4C). Besides, CCr was increased when treated with GIT1 (Figure 4D). Altogether, these data suggest that overexpression of GIT1 attenuates sFlt-1-induced preeclampsia symptoms.

### Additional GIT1 upregulates serum NO level and placenta eNOS activity

**Figure 5:**
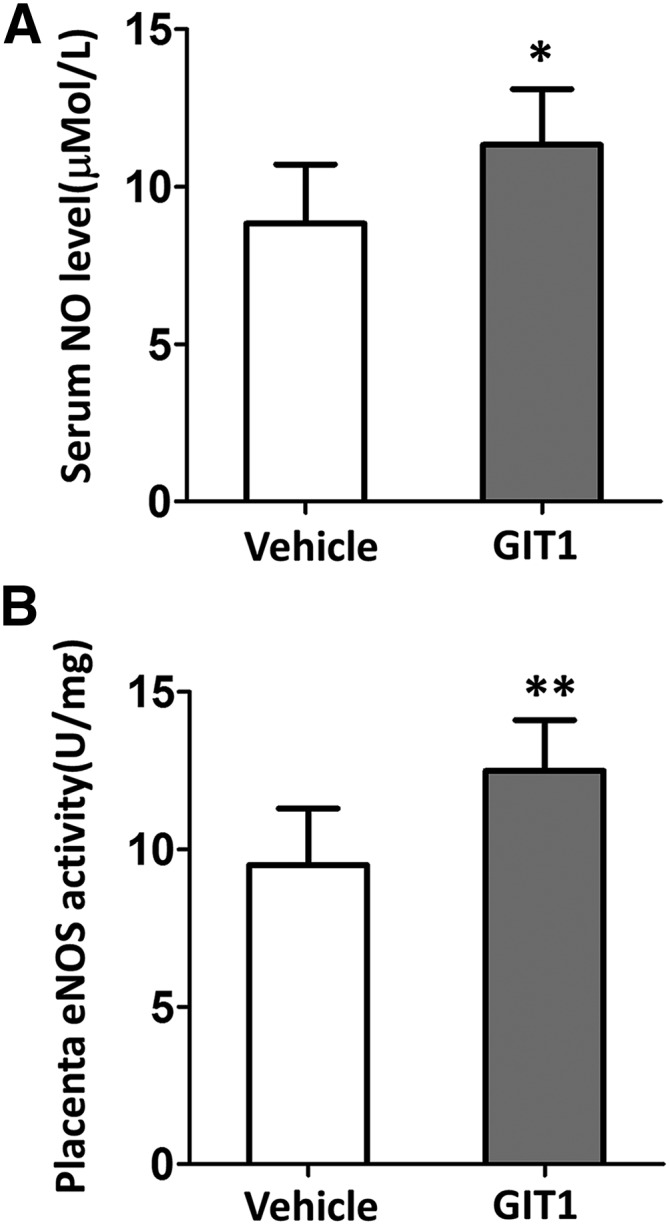
Additional GIT1 upregulated serum NO level and placenta eNOS activity. (A) Serum NO level (B) Placenta eNOS activity. The tests were done at day 6 after administration of sFlt-1/Fc(control). N = 6 mice in each experimental group. *P < 0.05, **P < 0.01 *vs.* Vehicle mice.

To further elucidate the specific mechanism of GIT1 in preeclampsia, serum NO levels and placenta eNOS activities were detected in WT and GIT1 overexpression mice induced by sFlt-1. Serum NO levels (Figure 5A) and eNOS activities (Figure 5B) were significantly increased when treated with additional GIT1 (*P* < 0.05 and *P* < 0.01, respectively). Our findings might molecularly explain the mechanism that GIT1 alleviates sFlt-1-induced preeclampsia symptoms by regulating NO production and eNOS activity.

## Discussion

Preeclampsia is a multisystem disorder of pregnancy and affects approximately 2–8% of overall pregnancies worldwide (US Preventive Services Task Force *et al.* 2017). Preeclampsia can be normally characterized by hypertension (the onset of high blood pressure) and proteinuria (a significant amount of protein in the urine) fter 20 weeks of gestation (de Groot and Taylor 1996). Accounting for approximately 16% of the direct maternal deaths, preeclampsia is a major cause of maternal mortality in developed countries, which might lead to a wide spectrum of serious complications, such as hemorrhagic stroke, elevated liver enzymes and low platelets (HELLP) syndrome, eclampsia, and renal failure and pulmonary edema (Lambert *et al.* 2014; Arulkumaran and Lightstone 2013). Although the etiology of preeclampsia stills remains to be further elucidated, it is widely accepted that the increased production of soluble fms-like tyrosine kinase 1 (sFlt-1), soluble endoglin, and likely other factors in placenta by placental hypoxia/ischemia accounts for the occurrence of preeclampsia (De Vivo *et al.* 2008). Previous studies have demonstrated that the increased expression of sFlt-1 is considered as one of the most critical factors of preeclampsia maternal symptoms (Carney 2015). Generally, pregnant women with preeclampsia possess higher level of plasma concentration of sFlt-1 compared with normal pregnant women, and this expression is expanded in severe preeclampsia women (Youssef *et al.* 2011). Thus, in our study, we used adenovirus to overexpress sFlt-1 for inducing preeclampsia in mice and explored the function of GIT1 in monitoring sFlt-1-induced preeclampsia phenotype.

Previous studies have proved that eNOS plays a crucial role in vascular homeostasis (Sandrim *et al.* 2010). A complex set of signaling and post-translational structural biological processes are involved in the regulation of eNOS function (Singh *et al.* 2010). For instance, eNOS has been found to be interact with various proteins, such as HSP-90, G-protein-coupled receptor kinase 2 (GRK2), caveolin-1, and notably, GIT1 (Niu and Qi 2011). These protein interactions generally result in changes in the activity and function of eNOS by modifying the phosphorylation of several key serine residues (Fatini *et al.* 2006). It has been reported that GIT1 significantly enhances the eNOS activity by direct interaction with eNOS protein in sinusoidal endothelial cells (Li *et al.* 2012). A simple model has been proposed that activated Akt could phosphorylate eNOS at Ser^1177^ to stimulate NO production (Dimmeler *et al.* 1999). However, recent studies have shown that eNOS could be phosphorylated by a relatively complex system. It has been found that the interaction between GIT1 and eNOS depends on the phosphorylation of specific residues in GIT1 (Tyr^293^ and Tyr^554^) (Watts and Motley 2009). A tightly linked system of regulatory events centered on the GIT1 scaffold was identified to regulate the activation of eNOS (Tanaka *et al.* 2015). Upon endothelin stimulation, GIT1 was phosphorylated on tyrosine residues, predominantly Tyr^293^ and Tyr^554^ in sinusoidal endothelial cells (Wu *et al.* 2014). Previous findings have demonstrated that Src kinase family are responsible for the phosphorylation of these two sites (Tanaka *et al.* 2015). Here, we extend these findings by exploring the function of GIT1 in regulating eNOS activity in sFlt-1–induced preeclampsia mice. In this paper, we demonstrate that depletion of GIT1 significantly impedes the NO production and placenta eNOS activity in mice (Figure 3). However, more detailed molecular mechanisms of how GIT1 regulates eNOS activity in preeclampsia mice still need to be further elucidated. In view of the fact described above, here we hypothesize that GIT1 might directly bind to eNOS in placenta, and phosphorylation of GIT1 is crucial in the GIT1-eNOS interaction and in stimulation of eNOS phosphorylation. Moreover, Src and Akt might both act upstream and downstream of GIT1 in the GIT1-eNOS pathway, leading to eNOS activation that we observed mediated through the GIT1 scaffold.

Previous studies have found that overexpression of sFlt-1 induces a preeclampsia–like phenotype, which includes increased BP, proteinuria, and endothelial dysfunction (Caillon *et al.* 2018). Also, it has been demonstrated that increased sFlt-1 will lead to a more severe preeclampsia-like phenotype in eNOS^−/−^ mice (Navaratnam *et al.* 2017). Symptoms including the increase of UAE, the decrease of CCr, and more severe endotheliosis are aggravated by a synergistic effect combining the overexpression of sFlt-1 and the depletion of eNOS.(Reddy *et al.* 2009) In addition, when overexpressed sFlt-1, eNOS^−/−^ mice has a higher renal expression of the ET system compared with the WT mice (Zhang *et al.* 2013). Preproendothelin-1 (ET-1) has been demonstrated to exacerbates the pathologic changes generated from the additional expression of sFlt-1 (Kamoi *et al.* 1990). Pregnant women with preeclampsia normally exhibit higher levels of plasma ET-1 (Boulanger and Luscher 1990). Since NO suppresses the expression level of ET-1, previous studies have proved that the decreased production of NO and the suppressed eNOS activity might exacerbate preeclampsia-like phenotypes by upregulating ET-1 (Liang *et al.* 1996). In addition, depletion of eNOS and increased sFlt-1 enhance the expression level of preproET-1 and ET_A_R in the kidney (Li *et al.* 2012). In this paper, we demonstrate that depletion of GIT1 significantly impedes the NO production and placenta eNOS activity (Figure 3). Thus, we can assume that GIT1 might be influential on the regulation of ET-1.

In conclusion, our data demonstrate that absence of GIT1 exacerbates the preeclampsia-like phenotypes induced by the overexpression of sFlt-1 in pregnant female mice. Depletion of GIT1 decreases BP and UAE, and increases CCr both in WT and sFlt-1 mice. In addition, GIT^−/−^ sFlt-1 mice exhibit reduced expression of NO production and decreased activity of eNOS. Overexpression of GIT1 demonstrates a reversed effect. Although future studies could be made to further reveal the specific molecular mechanisms of how GIT1 aggravates the sFlt-1–induced preeclampsia-like phenotype by regulating NO/eNOS pathway, our research provides reliable clues and basis for the follow-up study on preeclampsia.
